# Extraction of protein from apricot kernel oil press cake (AKOPC) through innovative techniques and the formulation of supplemented yogurt

**DOI:** 10.1002/fsn3.3544

**Published:** 2023-07-21

**Authors:** Rabbiya Choudhry, Adeela Yasmin, Muhammad Arslan Aslam, Ali Imran, Rabia Shabir Ahmad, Farhan Saeed, Fakhar Islam, Tahir Zahoor, Mohd Asif Shah, Adil Rasool

**Affiliations:** ^1^ Department of Food Science, Faculty of Life Sciences Government College University Faisalabad Pakistan; ^2^ Department of Clinical Nutrition NUR International University Lahore Pakistan; ^3^ School of Business Woxsen University Hyderabad India; ^4^ Division of Research and Development Lovely Professional University Phagwara India; ^5^ School of Engineering and Technology Sharda University Greater Noida India; ^6^ Department of Management Bakhtar University Kabul Afghanistan

**Keywords:** apricot kernel, protein extraction, skim milk powder, yogurt

## Abstract

The apricot kernel oil press cake (AKOPC) is a high protein natural by‐product of oil mechanical expression with potential uses in cosmetics, medicines, and food. The purpose of this research was to improve the protein extraction process from apricot kernel oil press cake by using enzymatic extraction (EEP), aqueous extraction (AEP), and ultrasound extraction (UEP) process. Protein extraction by AEP was facilitated by a low solid–liquid ratio (SLR) (1:15.97) and prolonged reaction durations (3.30 h), resulting in extraction yields of 68%. When compared to the AEP by similar reaction time, increased enzyme utilization (0.90%) in the EEP resulted in greater protein extraction yields (70%) in a shorter reaction time. In addition to AEP and EEP, ultrasound extraction was also used to improve protein extractability. Temperature (50°C), power density (225 W/L), and extraction duration (20 min) were shown to be the best extraction points. Protein yield was found to be 56.47% at ideal UEP conditions. The experimental values for these reactions were found to be equivalent to the predicted values formed by the mathematical models. When supplementary skimmed milk powder (SMP) was substituted with apricot kernel protein (AKP) in the yogurt manufacturing process, the total solids, average titratable acidity, total protein, and fat contents of the yogurt were increased. In contrast, pH and syneresis values decreased as AKP increased in the resulting yogurt, whether fresh or after 7 days of cold storage. Substitution of additional SMP with AKP in yogurt production might be recommended up to 35%.

## Graphical abstract

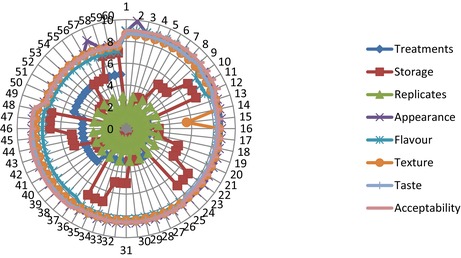

## 1 INTRODUCTION

The global demand for proteins is rising, necessitating the development of novel dietary protein sources. Vegetarians and health‐conscious consumers are increasingly interested in protein‐rich diets derived from plants that contain no cholesterol and have a low saturated fat content (Eyidemir & Hayta, 2009; Rasheed et al., 2023). As functional components in food compositions, vegetable proteins represent a cost‐effective and flexible alternative to animal proteins. In terms of market price, land required, and environmental effects, animal proteins are costly (Asghar et al., 2023; Rai et al., 2016). Significant protein, water, and energy losses occur during the conversion of vegetable protein to animal protein. The energy consumption per kilogram of animal protein is 8–10 times that of vegetarian protein under industrial conditions (Fatima et al., 2023; Gecer et al., 2020).

Additionally, growing raw material and energy prices are driving demand for low‐cost, high‐quality protein products. The apricot (*Prunus armeniaca* L.) belongs to the Rosaceae family and is a popular fruit worldwide. Apricot kernels are a by‐product of several food processing enterprises that use apricots and may be bought for a very low price. The oil extracted from apricot kernel is now utilized in a variety of sectors such as oil/fat, cosmetics, pharmaceuticals, and baking (Rampáčková et al., 2021). Upon oil extraction from kernels of apricot, about 60% of the leftover residue, known as press cake, contains almost 50% protein; therefore, it can be used as a protein source after being treated to remove the bitter component (Alajil et al., 2021). As a result, the high‐protein content of the kernel meal remaining after apricot kernel oil extraction may be used to make protein isolate after necessary detoxification. This protein can be utilized to supplement several food sectors with cheap and high‐quality vegetable protein (Wang et al., 2015).

Because the kernel meal contains the bitter and poisonous chemical hydrocyanic acids (HCN), it is necessary to detoxify the cake before extracting the isolate (Asghar et al., 2023; Dhen et al., 2018). The existence of large quantities of amygdalin in kernels of apricot prohibits them from being used as a food source, despite their high protein and carbohydrate content (Mahali & Sibi, 2019). This chemical has been advertised as cancer preventative despite its recognized toxicity (Sharma et al., 2010). Thus, water dilution of press cake at a ratio of 1:20 (w/v) after boiling for 60 min resulted in a free‐flowing slurry with a higher yield, which was suggested for detoxification of kernel meal before its usage for protein isolate extraction (Alimentaria, 2000).

Enzymatic extraction process (EEP) and aqueous extraction process (AEP) are ecologically acceptable methods for extracting protein from a variety of oil‐bearing materials without using neurotoxic and flammable hexane. In comparison with traditional procedures, ultrasound extraction process (UEP) has gained a lot of interest in the previous decade, especially for protein extraction (Abugabr Elhag et al., 2019). The challenge in the EEP, AEP, and UEP was to determine protein efficiency and to achieve high extraction yield (Alpaslan & Hayta, 2006).

The purpose of this research was to extract protein from AKOPC, which is an inexpensive and high‐protein food. To assess the sensory acceptability of protein‐fortified yogurt formulations and to improve protein consumption by the general public by adding it in yogurt, which is popular across all age groups. To my knowledge, no research has been done to investigate the extraction and supplementation of protein isolate from apricot kernel meal using these novel techniques.

## 2 MATERIALS AND METHODS

Apricot kernels of variety *Prunus armeniaca L* were procured from Ayub Agriculture Research Institute, Faisalabad, Pakistan. Milk and other consumables, as well as reagents such as starter culture and lactic acid bacteria (LAB), were purchased from the local market for product development. Reagents and standards were purchased from Merck (Merck KGaA) and Sigma‐Aldrich (Sigma‐Aldrich).

### 2.1 Apricot kernel oil press cake (AKOPC)

AKOPC was produced by cold pressing of grounded apricot kernels and passed through a sieve with a mesh size of 20. The chemical composition was analyzed using AOAC (2000) procedures for crude fat, moisture, crude fiber, ash, crude protein, and hydrocyanic acids (HCN) (AOAC, 2000).

#### 2.1.1 Moisture content

The moisture level of apricot kernel oil press cake was determined using the appropriate AOAC (2016) procedure. Therefore, a 10‐g sample was dried in Memmert oven (Model VO49) at a temperature of 105 ± 5°C for the duration until the weight was constant.

#### 2.1.2 Crude protein

Kjeltec apparatus was used to determine the nitrogen content of apricot kernel oil press cake in accordance with AOAC (2016). By multiplying nitrogen content by a conversion factor of 6.25, the protein percentage was calculated.

#### 2.1.3 Crude fat

The crude fat content present in apricot kernel oil press cake sample was measured by their respective AOAC (2016). Using n‐hexane as a solvent, 3 g of dried material was refluxed in a soxhlet apparatus (Model:H‐21045 Extraction Unit, Hoganas, Sweden).

#### 2.1.4 Crude fiber

The crude fiber content of apricot kernel oil press cake sample was measured using the AOAC technique (2016). In the Labconco Fibertech equipment, fat‐free sample was digested with 1.25% H₂SO₄ and then 1.25% NaOH solution (Labconco Corporation). And after filtering and washing with distilled water, the leftover residues were weighed and burned at 550–560°C in a muffle furnace until residues were recovered.

#### 2.1.5 Ash

The ash percentage of apricot kernel oil press cake sample was determined using the AOAC technique (2016). In a crucible, a 5‐g sample was directly burned on the flame until no fumes appeared. After that, the sample was burned at 550–600°C for 5–6 h in a muffle furnace (MF‐102, PCSIR) until a grayish residue was produced.

#### 2.1.6 HCN

With a small modification (Sharma et al., 2010), technique for extracting hazardous compounds from apricot kernels was used. The apricot kernels were soaked in water overnight at 40°C (1:12 w/v ratio). In a Memmert vacuum oven (Model VO49), the entire slurry was dried overnight at 40°C. Fats and lipids were extracted many times with hexane after drying. The defatted meal was air‐dried and ground to pass through a 60‐mesh sieve at room temperature (25°C). For analysis, the apricot kernel flour was kept at 4°C.

After detoxification, the sample was taken to the laboratory and evaluated directly. A random selection of 100 g of each sample was made. The sample was separated into small pieces (20–30 mm) and dried in a Memmert vacuum oven (Model VO49) at 60°C for 12–24 h until it achieved an even dry mass. Before extraction, the dried material was pressed with a laboratory grinder to a particle size of less than 1 mm.

### 2.2 Aqueous extraction process (AEP) of apricot kernel oil press cake

To produce AKOPC‐to‐water ratios ranging from 1:05.18 to 1:90.12, about 50 g of apricot kernel oil press cake was dispersed in water. Extractions were carried out at 45°C and pH 9.0, with reaction durations ranging from 1 to 4 h and continuous stirring at 120 rpm (Balvardi et al., 2015; Pojić et al., 2018).

The primary data were used to choose the pH of the reaction. After extraction, the slurry was centrifuged at 3000*g* for 25 min at 30°C to separate the insoluble fraction from the liquid fraction, which was then frozen at −20°C. All protein extracted from the apricot kernel oil press cake (AKOPC) was determined.

The protein was analyzed. The recommended extraction conditions were tested in three different ways. Approximately 50 g of the protein was freeze‐dried using a Zirbus Technology Benelux B.V freeze drier available in VaCO_2_ and VaCO_5_ at Government College University Faisalabad kept at −20°C for further solubility testing.

### 2.3 Enzymatic extraction process (EEP) of apricot kernel oil press cake

In the EEP of kernel meal, protease‐N (EC 3.4.24.28, Bacillus subtilis) was employed. The primary data were used to choose the enzyme. The effects of the AKOPC‐to‐water ratio (1:12.14 to 1:90.12) and the quantity of enzyme (0.20%–0.90%) on protein extraction yields in the EEP were investigated individually and in combination (Moura et al., 2009).

Extraction was carried out by dispersing 50 g of AKOPC in water at a solids‐to‐liquid ratio of 1:12.14 to 1:90.12. Prior to actually adding 0.20%–0.90%t enzyme (weight/weight of apricot kernel cake), the slurry was adjusted to pH 9.0, and extraction was carried out at 45°C with consistent stirring at 120 rpm. At 1‐h, extraction kinetics were assessed.

The slurry was centrifuged at 3000*g* for 25 min at 30°C after extraction to separate the insoluble fraction from the liquid fraction. For further examination, the fractions were kept at −20°C. According to the equation specified in the AEP, the total protein extraction yield was determined.

The protein was analyzed. The recommended extraction conditions were tested in three different ways. Approximately 50 g of the protein was freeze‐dried using a Zirbus Technology Benelux B.V freeze drier available in VaCO_2_ and VaCO_5_ at Government College University Faisalabad and kept at −20°C for later solubility testing.

### 2.4 Ultrasound extraction (UEP) of apricot kernel oil press cake

Apricot kernel protein extraction was carried out according to the method outlined by Song et al. (2013) with a few modifications (Song et al., 2013). In 1000 mL of deionized water, 50 g of apricot kernel meal was distributed. Using 1 mol/L NaOH at room temperature for 1 h, the pH of the mixture was adjusted to 8.0. The mixture was centrifuged for 20 min at 4500*g* after ultrasonic treatments. After correcting the pH of the supernatant to 4.5 with 1.0 mol/L HCl, the protein isolate was produced by centrifuging at 4500*g* for 20 min. Deionized water (pH 4.5) was used to wash the precipitates twice. The precipitate was suspended again in deionized water with 0.1 mol/L NaOH added to bring the pH to 7.0. To test the functional and physicochemical characteristics of Apricot kernel protein (AKP) under optimal conditions, the optimized protein isolate was dried using a freeze dryer and kept at −18°C.

### 2.5 Functional properties of protein isolate

Protein isolate was analyzed for physicochemical and functional properties like water‐holding capacity, oil holding capacity, protein solubility, emulsification properties, and foaming properties using the following procedures:

#### 2.5.1 Water absorption capacity

It was calculated by combining 1‐g sample with 20‐mL distilled water in centrifuge and then agitated for 50 s. The dispersions were agitated at 3500 *g* for 30 min after standing at room temperature. Whatman No. 1 filter paper was used to filter the supernatant and the volume was calculated precisely. Initial volume of the distilled water in the sample and the volume obtained after filtration were compared. The results were presented in milliliters of water per gram of material.

#### 2.5.2 Oil absorption capacity

It was calculated by inserting 1‐g sample into 15‐mL centrifuge tube. It was combined with 10‐mL refined groundnut oil by using agitator. Thirty minutes were given for samples to stand. Centrifugation of the sample‐oil combination took 25 min at 3500 *g*. After centrifugation, the supernatant was slowly poured into a 10‐mL cylinder and the volume (V2) was noted.

$$\text{Oil Absorption Capacity}=\frac{V1-V2}{W0}$$

#### 2.5.3 Emulsification capacity/activity

It was determined by homogenizing 5 g of sample in 50‐mL distilled water for 30 s at about 8000 *g*. After that, corn oil was added and mixture was homogenized again for 30 s. The emulsion was split into two equal volumes. Centrifugation was carried out at 1000 *g* for 10 min and then both volumes were heated to about 85°C for 20 min. To determine emulsion activity, the ratio of emulsion height to liquid layer height was noted.

#### 2.5.4 Foaming capacity

By mixing 50 mL of 3% (w/v) sample in distilled water and immediately pouring it into a graduated cylinder, foams' capacity was measured. The volume was noted both before and after whipping. The proportion of volume that was created by whipping is used to represent the foaming capacity.

#### 2.5.5 Protein solubility

One‐gram sample was homogenized in 25 mL of 0.5 M NaCl at pH 7 for about 60 min and then centrifuged at 8000 *g* for 30 min. The nitrogen content was determined in the soluble fraction. Solubility was the ratio of the original sample's total nitrogen to the soluble fraction.

### 2.6 Product development

Fat‐standardized buffalo milk (3.5% fat) samples from Agriculture University dairy farms were examined. It was tested for acidity, pH, solid‐not‐fat (SNF), protein, and milk fat tests. The AOAC (2016) procedure was used to examine all parameters. Conventional recipe was used.

Milk was standardized at 3.5% fat, 15%–18% total solids, and 2% sugar was added. Skimmed milk was also added in powder form, its composition was lactose 51%, protein 34%, mineral 8.2%, protein 34%, moisture 4%, and fat 1.2%. The control yogurt was prepared using fat‐standardized buffalo milk containing 3% fat and 1.5% skimmed milk powder (SMP) to enhance the consistency of the yogurt. All of the samples were pasteurized for 15 s at 72°C. Lactic acid culture was inoculated. The supplementary skimmed milk powder was substituted with protein isolate from apricot kernel meal at concentrations of 15%, 25%, 35%, and 45% (w/vol). Standardized milk was inoculated and placed into yogurt cups. The sample was kept in an incubator at 37–42°C for 4–5 h to achieve the necessary acidity level (Toffanin et al., 2015). For proximate analysis, the produced yogurt was stored in the refrigerator. Yogurt was then evaluated for pH, acidity, crude protein, crude fat, total solids syneresis, and viscosity.

### 2.7 Sensory analysis

Ten trained panel members assessed supplemented yogurt samples for sensory features such as texture, flavor, appearance, and general acceptability. Products were rated on a 9‐point Hedonic scale using consistent descriptive terms ranging from 9 “like extremely” to 1 “dislike extremely”.

### 2.8 Statistical analysis

The obtained data were analyzed statistically by using ANOVA (analysis of variance technique) to evaluate the statistical significance of treatments in Microsoft Excel 2016 and (Statistics 9.1). Duncan's multiple range test was used for mean separation (Wichchukit & O'Mahony, 2015).

## 3 RESULTS AND DISCUSSION

Chemical composition of AKOPC is demonstrated in Table 1. It was discovered that apricot cake contains 8.87%, 9.47%, 33.50%, 2.65%, 9.43%, and 0.74% moisture, crude fat, crude protein, ash crude fiber, and HCN, respectively. Sharma et al. (2010) had very identical findings, reporting 7.2% moisture, 34.3% protein, 9.7% crude lipids, 10.8% crude fiber, 27.5% carbs, and an intrinsic HCN level of 90 mg/100 g press cake. The small difference in data might be due to changes in weather circumstances, cultivar type, and stage of ripeness during harvesting, which could have altered the quality metrics.

**TABLE 1:** Physicochemical composition of apricot kernel oil press cake (AKOPC).

Chemical constituent	Observations (mean ± SE)
Moisture (%)	8.87 ± 0.02
Crude protein (%)	33.50 ± 0.20
Crude fat (%)	9.47 ± 0.10
Crude fiber (%)	9.43 ± 0.15
Ash (%)	2.65 ± 1.10
HCN (mg/100 g)	0.74 ± 0.06

### 3.1 Extraction of protein

#### 3.1.1 Aqueous extraction process of apricot kernel oil press cake: Process optimization

**GRAPH 1:**
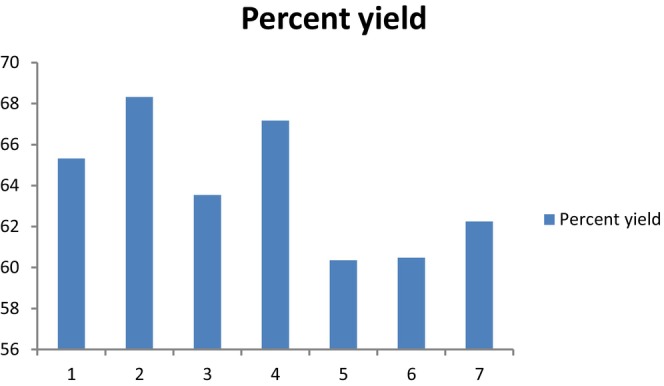
Effect of AKOPC‐to‐water ratio and reaction time on aqueous extraction yield. Experimental Conditions: 1 (1:90 AKOPC‐to‐water ratio, 90 min); 2 (1:15 AKOPC‐to‐water ratio, 210 min); 3 (1:90 AKOPC‐to‐water ratio, 210 min); 4 (1:15 AKOPC‐to‐water ratio, 150 min); 5 (1:11 AKOPC‐to‐water ratio, 150 min); 6 (1:05 AKOPC‐to‐water ratio, 60 min); 7 (1:05 AKOPC‐to‐water ratio, 240 min); 8, 9, 10, and 11 central points (1:05 AKOPC‐to‐water ratio, 150 min).

The protein extractability is significantly influenced by processing factors such as the solids‐to‐liquid ratio, particle size, pH, temperature, and reaction duration. Graph 1 shows the outcomes of reaction time (1–4 h) and AKOPC‐to‐water ratio (1:05 to 1:90) on total protein extraction. Total protein extraction ranged from 60.4% to 68.3%, with the use of low AKOPC‐to‐water ratio being preferred. The larger gradient concentration between the aqueous medium and solutes when low AKOPC‐to‐water ratio is utilized favors protein solubilization and following diffusion into the aqueous medium, which is the likely cause of the better protein extractability found at low AKOPC‐to‐water ratio. Reduced extraction medium viscosity at lower AKOPC‐to‐water ratio should be anticipated, facilitating protein diffusion into the aqueous medium.

Our findings are consistent with a number of papers in the literature (Moura et al., 2009) that found that attempting a greater AKOPC‐to‐water ratio generally reduced the protein extractability in the AEP.

Validation experiments were carried out using the lowest AKOPC‐to‐water ratio evaluated (1:15.97) at three reaction times (1:30, 2:30, and 3:30 h) in order to maximize the simultaneous extraction of protein. To evaluate the effectiveness of the predictive models, this experimental condition was carried out three times. At 45°C and pH 9.0 with constant agitation at 120 rpm, extractions were conducted.

Results show that to maximize protein extraction at pH 9.0 and 45 C, a low AKOPC‐to‐water ratio (1:15.97) and 2.30 h of reaction duration is sufficient. The apricot kernel cake can be processed to remove 70% of its protein under these circumstances. Under these conditions, 70% of protein can be obtained from the apricot kernel meal. Comparing our findings to the literature is difficult. A few results were obtained by Bildstein et al. (2008) who utilize aqueous extraction approach with glucoamylase to obtain food‐grade protein isolates from white beans and lentils. In another research, Wang et al. (2008) used this method for the extraction of protein and oil hydrolysates from peanut.

#### 3.1.2 Enzymatic extraction process of apricot kernel oil press cake: Processing optimization

The extent to which enzymatic hydrolysis increases the yields of protein extraction from numerous oil‐bearing substances depends on the kind and quantity of enzymes utilized as well as the thermal and mechanical treatments that the sample had initially undergone. The individual and combined impacts of AKOPC‐to‐water ratio (1:12.14 to 1:90.12) and enzyme concentration (Protease‐N) (EC 3.4.24.28, Bacillus subtilis) (0.20%–0.90%) on protein extractability was assessed in order to evaluate the efficiency of utilizing proteases during the extraction process.

The percentage of total protein extraction ranged from 60.21% to 70.01%. Regardless of the extraction duration, total protein extraction (TPE) was preferred by employing a low AKOPC‐to‐water ratio and a higher quantity of enzyme. Almost AKOPC‐to‐water ratio of 1:15.82 can be utilized to increase the total extractability of 75% protein from the apricot kernel meal. Extractions were carried out at 45°C, pH 9.0, under continuous stirring at 120 rpm for about 1 h. All experimental conditions were validated in triplicate to ensure the accuracy of the predicted models. When the reaction time was enhanced from 1 to 2 h, increase in TPE was not seen. Our findings are in accordance with those in the literature (Moura et al., 2009), which associate higher protein extractability with the conversion of proteins into much more soluble peptides by proteases.

According to the results of this research, protein extraction can be increased at shorter reaction times by using a low AKOPC‐to‐water ratio (1:15.82) and more enzyme (0.90% wt/wt) (i.e., 1 h). At 45°C, pH 9.0, and continuous stirring at 120 rpm for 1 h, extractions were carried out. To ensure that the predictive models were adequate, each experimental condition was evaluated three times. As discussed in the AEP section, comparing our results to the literature is difficult due to the lack of research reporting yields of protein extraction from apricot kernel cake produced by mechanical pressing shown in 2, 3, 4.

A study by Moura et al. (2009) on soybeans and cream de‐emulsification for the extraction of protein and oil shows similar results. Two commercial endoproteases were used for extraction. In another study, Li et al. (2006) extracted phenolics from citrus peels and evaluated using Folin–Ciocalteu assay.

**GRAPH 2:**
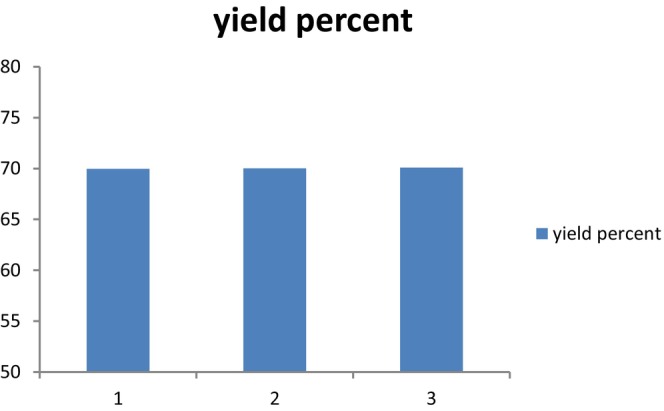
Experimental Validation of AEP conditions. Experimental validation of the AEP conditions: (1:15.97 AKOPC‐to‐water ratio at 90, 150, and 210 min).

**GRAPH 3:**
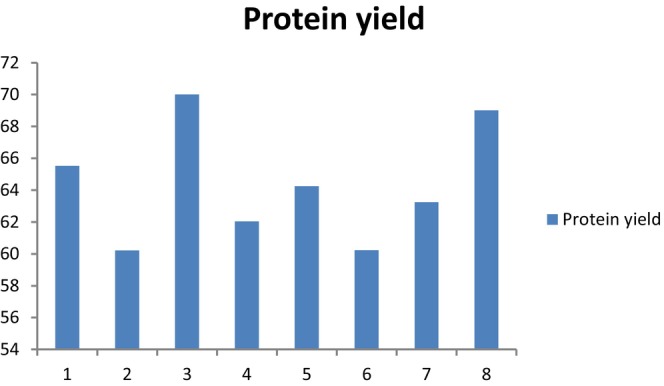
Effect of AKOPC‐to‐water ratio and enzyme amount on extraction yields generated by EEP. Experimental Conditions: 1 (1:15 AKOPC‐to‐water ratio and 0.30% protease); 2 (1:90 AKOPC‐to‐water ratio, 0.30% protease); 3 (1:15 AKOPC‐to‐water ratio, 0.80% protease); 4 (1:90 AKOPC‐to‐water ratio, 0.80% protease); 5 (1:15 AKOPC‐to‐water ratio, 0.55% protease); 6 (1:80 AKOPC‐to‐water ratio, 0.55% protease); 7 (1:12 AKOPC‐to‐water ratio, 0.20% protease); 8 (1:12 AKOPC‐to‐water ratio, 0.90% protease); 9, 10, and 11 (AKOPC‐to‐water ratio 1.12, 0.55% protease).

**GRAPH 4:**
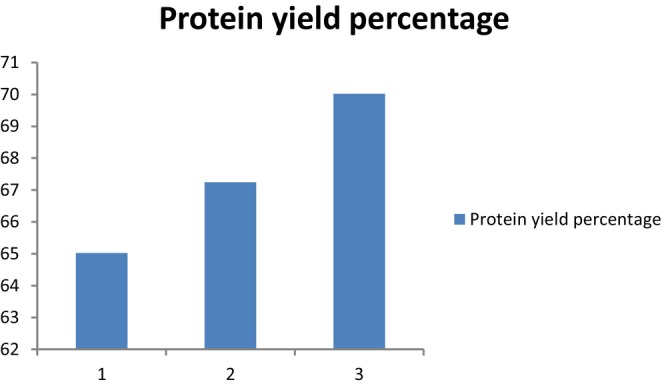
Experimental validation of the EEP conditions. Experimental validation of the EEP conditions: (1:15.82 AKOPC‐to‐water ratio and 0.20%, 0.55%, and 0.90% protease (wt/wt of apricot kernel oil press cake).

#### 3.1.3 Ultrasound extraction process of apricot kernel oil press cake

A sonication apparatus (Model VCX 750, Sonic and Materials, Inc) was used to explore the effect of power density, temperature, and time on protein yield. The graph shows the protein yield (%) achieved after 15 runs, as well as the actual and predicted values for Y1. The maximum protein yield (56.47%) was obtained via the implementation of 225 W/L, 50°C, and 20 min. and It was found that the combination of 225 W/L, 50°C, and 20 min produced the highest yield value (56.47%).

When compared to the conventional extraction method used by Saini et al. (2021), the protein percentage at the optimal extraction conditions of ultrasonic treatments was greater. In addition, the protein percentage under the ideal conditions was enhanced significantly by 4.35% and 7.30% as compared to ultrafiltration and isoelectric precipitated methods, respectively, for protein extracts from dehulled flour of green lentils. When compared to protein extracts from kabuli chickpea dehulled flour and desi chickpea dehulled flour, respectively, the protein yield increased significantly at the optimal ultrasonic conditions by 7.30% and 1.30%. These results were obtained using the isoelectric precipitated and ultrafiltration techniques. In comparison with the control and other technologies, the results showed that ultrasound treatment could enhance the protein yield from apricot kernel oil press cake.

**GRAPH 5:**
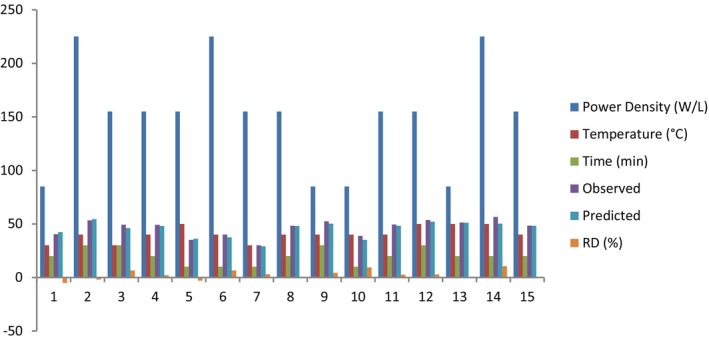
Ultrasound‐assisted extraction apricot kernel protein. Relative deviation (RD) = ((measured value ‐predicted value)/measured value) × 100.

These findings were consistent with those obtained by Roselló‐Soto et al. (2014), who extracted protein from olive kernel by UE method and high voltage electrical discharges and pulsed electric field were used as a pretreatment. In another study, Zhu and Fu (2012) evaluated the protein extraction from perilla seed cake and (Dabbour et al., 2018) from sunflower meal. Results show that highest yield was obtained from enzyme‐assisted extraction process shown in Graph 5.

### 3.2 Physicochemical characteristics of protein isolate

Regarding the physicochemical characteristics of protein isolate, Table 2 reveals the following information. The moisture content was 10.22, and the ash value was 3.17. Soluble protein, crude protein, carbohydrate, and crude fiber were observed to be 12.85, 90.46, 8.64, and 1.42, respectively. The crude fat and water activity were calculated to be 0.62 and 0.77, respectively. However, due to detoxification, the toxin hydrocyanic acid was discovered to be nil. The findings were consistent with the results of Thakur et al. (2019). They stated that apricot protein isolate has 12.67% moisture in it. The water activity, however, was 0.88. There were measurements of 5.84% total ash, 90.15% crude protein, 14.65% crude fiber, and 0.21% crude fat, respectively. The same outcomes were reported by El‐Aal et al. (1986) (Table 3).

**TABLE 2:** Physicochemical properties of apricot kernel protein isolate.

Parameters	Observations (mean ± SE)
Moisture (%)	10.22 ± 2.12
Soluble protein (%)	12.85 ± 1.38
Crude protein (%)	90.46 ± 1.45
Water activity	0.77 ± 0.10
Crude fiber (%)	1.42 ± 1.06
Total ash (%)	3.17 ± 2.53
Crude fat (%)	0.62 ± 0.41
Hydrocyanic acid, mg/100 g	Not detected
Carbohydrate (%)	8.64 ± 3.67

**TABLE 3:** Functional properties of apricot kernel protein isolate.

Parameters	Observations (mean ± SE)
Oil absorption Capacity (g/g)	1.86 ± 0.58
Water absorption capacity (g/g)	1.51 ± 0.90
Emulsion activity/Capacity (%)	50.02 ± 2.01
Foaming capacity (%)	22.30 ± 2.64
Protein solubility (%)	87.33 ± 2.08
Foaming stability (%)	45.74 ± 1.32

### 3.3 Functional properties of protein isolate

The physical and chemical characteristics of a protein which affect how it behaves in food systems during preparation, processing, storage, and consumption are known as its functional characteristics. The ability of food components to absorb water and oil is a crucial functional property since it enhances the mouthfeel and taste retention of meals. Protein may interact with water and oil in meals because it has both hydrophilic and hydrophobic characteristics. Conversely, protein isolates with high water absorption play a role to minimize moisture loss in packaged goods. Functional characteristics of protein isolate were observed to be 1.86 g and 1.51 g for oil absorption and water absorption capacity, respectively. Emulsion capacity, foaming capacity, protein solubility, and foaming stability were 50.02%, 22.30%, 87.33%, and 45.74%, respectively. Our findings support the prior conclusions of Thakur et al. (2019). According to their claims, protein isolate has a 2.45 mL/g water absorption capacity, a 2.52 mL/g oil absorption capacity, a 52.00% emulsifying capacity, an 88.00% protein solubility, and a 20.00% foaming capacity. The same outcomes were reported by El‐Aal et al. (1986).

### 3.4 Physicochemical analysis of supplemented yogurt

Yogurt was made with buffalo milk and skimmed milk powder was partially replaced with apricot kernel protein isolate obtained by an extraction process. This supplemented yogurt was further tested by chemical techniques such as pH, acidity, protein, total solids, fat, and sensory analysis, which were calculated for supplemented yogurt.

#### 3.4.1 Flow sheet of yogurt processing

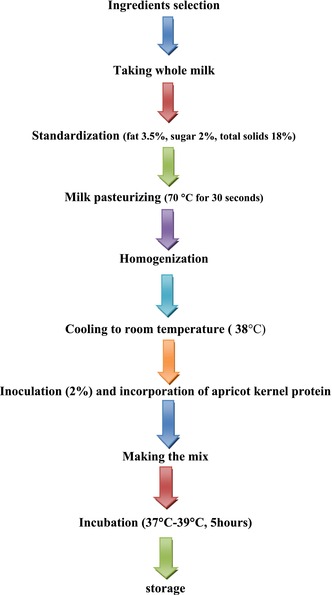

Table 4 shows the physicochemical characteristics of yogurt that has been enriched with various amounts of apricot kernel protein. The pH and titratable acidity readings for the yogurt samples ranged from 3.64 to 4.68 and 0.66% to 1.38%, respectively. During the storage period, the pH values dropped while the titratable acidity rates increased. These results support the claims made by Elkot et al. (2017) that shelf life lowers pH and raises titratable acidity. Dissociated lactic acid is produced by lactose fermentation during the shelf life, which raises the acidity level and lowers the pH (Brückner‐Gühmann et al., 2019). Additionally, the pH variation throughout the storage period is determined by the caseins' ability to act as a buffer. Yogurt's inclusion of plant protein had no obvious effect on the pH levels (*p* > .05).

**TABLE 4:** Effect of partial replacement of SMP by apricot kernel protein on chemical properties of yogurt.

SMP replacement level with AKP.	Storage time (days)	pH	Crude fat (%)	Crude protein (%)	Acidity (%)	Total soluble solids (%)	Syneresis (%)	Viscosity (g.s)
0	0	4.50 ± 0.22	7.15 ± 0.20	3.44 ± 0.17	0.73 ± 0.03	12.24 ± 0.61	30.45 ± 1.52	83.02 ± 1.66
	3	4.21 ± 0.21	7.14 ± 0.35	3.57 ± 0.17	1.11 ± 0.05	12.73 ± 0.63	30.43 ± 1.52	86.17 ± 1.72
	5	3.64 ± 0.18	7.22 ± 0.36	3.67 ± 0.18	1.23 ± 0.06	13.01 ± 0.65	30.36 ± 1.51	87.65 ± 1.75
	7	3.86 ± 0.19	7.22 ± 0.36	3.76 ± 0.18	1.38 ± 0.06	13.01 ± 0.65	30.32 ± 1.51	89.09 ± 1.78
15	0	4.62 ± 0.23	7.34 ± 0.36	3.84 ± 0.19	0.69 ± 0.03	12.42 ± 0.62	27.23 ± 1.36	94.23 ± 1.88
	3	4.31 ± 0.21	7.34 ± 0.36	3.86 ± 0.19	0.87 ± 0.04	12.83 ± 0.64	27.17 ± 1.35	96.49 ± 1.92
	5	4.14 ± 0.20	7.37 ± 0.36	4.04 ± 0.20	1.04 ± 0.05	12.93 ± 0.64	27.14 ± 1.35	96.84 ± 1.93
	7	4.03 ± 0.20	7.37 ± 0.36	4.06 ± 0.20	1.05 ± 0.05	13.03 ± 0.65	26.97 ± 1.34	97.24 ± 1.94
25	0	4.66 ± 0.23	7.71 ± 0.38	4.17 ± 0.20	0.79 ± 0.03	13.03 ± 0.65	25.25 ± 1.26	98.33 ± 1.96
	3	4.30 ± 0.21	7.75 ± 0.38	4.24 ± 0.21	0.87 ± 0.04	12.82 ± 0.64	25.20 ± 1.26	99.45 ± 1.98
	5	4.11 ± 0.20	7.75 ± 0.38	4.33 ± 0.21	1.04 ± 0.05	13.02 ± 0.65	25.12 ± 1.25	101.08 ± 2.02
	7	3.86 ± 0.19	7.71 ± 0.38	4.33 ± 0.21	1.20 ± 0.06	13.23 ± 0.66	24.96 ± 1.24	102.23 ± 2.04
35	0	4.66 ± 0.23	7.82 ± 0.39	4.56 ± 0.22	1.20 ± 0.06	13.0 ± 0.65	24.23 ± 1.21	106.83 ± 2.13
	3	4.39 ± 0.21	7.86 ± 0.39	4.65 ± 0.22	0.90 ± 0.04	13.05 ± 0.65	24.17 ± 1.20	108.48 ± 2.16
	5	4.13 ± 0.20	7.84 ± 0.39	4.68 ± 0.23	0.94 ± 0.04	13.14 ± 0.65	24.16 ± 1.20	109.01 ± 2.18
	7	3.94 ± 0.19	7.93 ± 0.39	4.73 ± 0.23	0.94 ± 0.04	13.24 ± 0.66	23.08 ± 1.15	109.37 ± 2.18
45	0	4.68 ± 0.23	8.17 ± 0.40	4.75 ± 0.23	0.66 ± 0.03	13.04 ± 0.65	23.04 ± 1.15	111.10 ± 2.22
	3	4.27 ± 0.21	8.12 ± 0.40	4.82 ± 0.24	0.66 ± 0.03	13.17 ± 0.65	23.03 ± 1.15	112.41 ± 2.24
	5	4.27 ± 0.21	8.19 ± 0.40	4.85 ± 0.24	0.66 ± 0.03	13.23 ± 0.66	22.98 ± 1.14	112.92 ± 2.25
	7	4.02 ± 0.20	8.23 ± 0.41	4.85 ± 0.24	0.66 ± 0.03	13.33 ± 0.66	22.07 ± 1.10	113.70 ± 2.27

Titratable acidity is important for sensory characteristics. Table 4 shows that on the first day of storage, samples with 25% apricot kernel protein added had the greatest titratable acidity (1.20%), whereas samples with 15% protein added had the lowest (0.69%). The added plant protein slows down the yogurt bacteria's proteolytic activity, increasing the synthesis of lactic acid, which results in the overall enhanced pattern of titratable acidity. A relevant study showed that the inclusion of ingredients that reduce proteolytic activity, such as mint and dill, raises yogurt's titratable acidity (Dubey & Kumari, 2011).

Syneresis is an essential structural characteristic of yogurts (Yildiz‐Akgül, 2018). The casein network rearrangements that occur during storage are often the cause of the serum separation, which is classified as a quality failure (Dubey & Kumari, 2011). The results showed that there were no significant differences between any of the control samples. As the amount of protein and storage days increase, the syneresis levels steadily decline.

A relevant study on the use of grape extract in the processing of cheese highlighted the possibility that hydrophobic bonding might be the reason in the present case (Brückner‐Gühmann et al., 2019). A reduction in serum separation during storage is also expected due to the starting culture's metabolic activities and the loss of the protein matrix's net pressure (Elkot et al., 2017).

#### 3.4.2 Sensory analysis

The spider graph demonstrates the sensory evaluation of yogurt in which specified quantities of skimmed milk powder were substituted with apricot kernel protein. Generally, the sensory scores of these AKP‐supplemented yogurts were not substantially different from sensory scores of the control yogurt and were all satisfactory. Although an increase in apricot kernel protein (AKP) concentration affects the appearance of the yogurt, it has no influence on the overall sensory ratings of the yogurt. Similar findings were published by Delikanli and Ozcan (2017).

**GRAPH 6:**
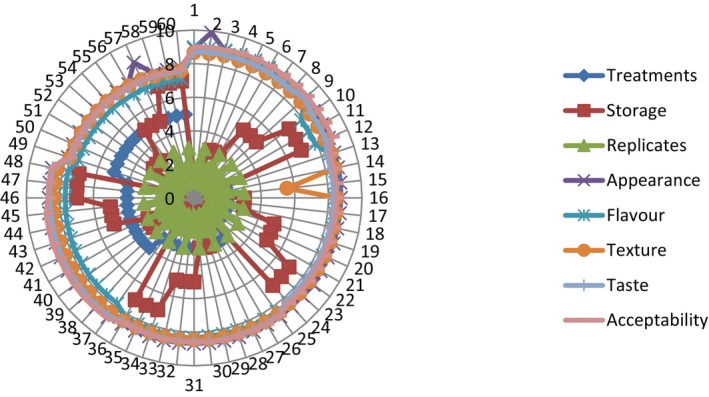
Sensory evaluation of yogurt made by replacing skimmed milk powder (SMP) with different ratios of apricot kernel protein.

The texture of the AKP‐supplemented yogurt improved as the AKP concentration rose (Graph 6). The flavor of the yogurt did not alter significantly as the concentration increased, demonstrating that the addition of AKP did not result in unwanted flavor changes (Elkot et al., 2017).

## 4 CONCLUSION

According to the current findings, the enzyme‐assisted aqueous extraction process produced the highest yield, ranging from 60.21% to 70.01%, while the ultrasound‐assisted extraction process produced 30.01%–56.47% and the aqueous extraction procedure produced 60.4% to 68.3%. It may also be concluded that an acceptable appetizing yogurt might be produced from a blend of buffaloes' milk (3.5% fat) by replacing 15%–35% supplementary skimmed milk powder with apricot kernel protein.

## AUTHOR CONTRIBUTIONS

**Rabbiya Choudhry:** Writing – original draft (equal). **Adeela Yasmin:** Supervision (equal); writing – review and editing (equal). **Muhammad Arslan Aslam:** Validation (equal). **Ali Imran:** Formal analysis (equal). **Rabia Shabir Ahmad:** Validation (equal). **Farhan Saeed:** Visualization (equal). **Fakhar Islam:** Methodology (equal). **Mohd Asif Shah:** Formal analysis (equal). **Tahir Zahoor:** Validation (equal). **Adil Rasool:** Software (equal).

## FUNDING INFORMATION

The authors declare that no funds, grants, or other support were received during the preparation of this manuscript.

## CONFLICT OF INTEREST STATEMENT

The authors declare that they have no conflict of interest.

## ETHICS STATEMENT

This article does not contain any studies with human participants or animals performed by any of the authors.

## CONSENT TO PARTICIPATE

Corresponding author and all the co‐authors are willing to participate in this manuscript.

## CONSENT FOR PUBLICATION

All authors are willing for the publication of this manuscript.

## Data Availability

Even though adequate data have been given in the form of Tables and Figures, all authors declare that if more data are required then the data will be provided on a request basis.
